# Accuracy of Umbilical Cord Complete Blood Count in Detecting Early-Onset Neonatal Thrombocytopenia

**DOI:** 10.7759/cureus.50503

**Published:** 2023-12-14

**Authors:** Mohammed Y Al-Hindi, Sherin A Qari, Wed A Fatani, Raneem M Alshaban, Nooran S Felemban, Jood M Altowairqi, Mansour A AlQurashi

**Affiliations:** 1 Pediatrics, King Abdulaziz Medical City, Ministry of National Guard Health Affairs, Western Region, Jeddah, SAU; 2 Research and Development, King Abdullah International Medical Research Center, Jeddah, SAU; 3 College of Medicine, King Saud Bin Abdulaziz University for Health Sciences, Jeddah, SAU

**Keywords:** diagnostic test accuracy, complete blood count, umbilical cord, specificity, sensitivity, thrombocytopenia, neonatal

## Abstract

Background: Neonatal thrombocytopenia (NTCP) is a common hematological disorder whose platelet count falls below the normal limit of 150 x 10^9^/L. NTCP can cause late complications if left untreated. The current study aimed to evaluate the accuracy of the umbilical cord complete blood count (UC CBC) in detecting early-onset neonatal thrombocytopenia (EO-NTCP). Further, the prevalence of NTCP was also investigated.

Methods: A cross-sectional study with a matched control was conducted on all newborns delivered at a tertiary care center in Jeddah, Saudi Arabia, between May 2016 and 2019. After exclusions, 40 neonates with EO-NTCP (cases) and 80 without EO-NTCP (controls) were included. The case-to-control ratio was 1:2. The results of UC CBC were compared with those of follow-up CBC, performed within 72 hours. A p-value of <0.05 was considered statistically significant. All data were analyzed using IBM SPSS version 28 for Windows (IBM Corp., Armonk, NY).

Results: The prevalence of NTCP was approximately 1.02% (111/10,936). Lack of antenatal care was found in 12 (30%) neonates with EO-NTCP vs. 10 (12.5%) neonates without EO-NTCP (p = 0.02). Neonates with EO-NTCP were more likely to have experienced intrauterine growth restriction (5 (37.5%) vs. 5 (6.3%), p < 0.001) and oligohydramnios (5 (12.5%) vs. 0 (0%), p = 0.003). Neonates who developed EO-NTCP were more likely to be admitted to the NICU (34 (85%) vs. 35 (43.8%), p < 0.001) and receive antibiotics (22 (55%) vs. 25 (31.3%), p = 0.012). Also, neonates with EO-NTCP were more frequently diagnosed with neonatal sepsis (7 (17.5%) vs. 3 (3.8%), p = 0.015) and more likely to receive platelet transfusions (15 (37.5% vs. 1 (1.3%), p < 0.001). They also had a higher median length of hospital stay (13 (interquartile range (IQR) 3-28) vs. 4 (IQR 2-9) days, p = 0.006). The mortality rates of neonates with EO-NTCP and those without were 6 (15%) vs. 2 (2.5%) neonates (p = 0.016). The sensitivity, specificity, positive predictive value (PPV), and negative predictive value (NPV) of UC CBC were 62.50%, 97.50%, 20.40%, and 99.61%, respectively.

Conclusion: The prevalence of EO-NTCP in King Abdulaziz Medical City is comparable to international and national figures, and it is associated with preceding maternal comorbidities, serious neonatal morbidity, and even mortality. Therefore, proper antenatal care is vital in preventing maternal and neonatal morbidities, including the risks of NTCP and its related complications. With high NPV, using UC CBC as a universal screening method could assist in safely discharging newborns. However, because of its low sensitivity, a comprehensive clinical examination with confirmatory laboratory tests are still the cornerstone in diagnosing EO-NTCP. Future trials should aim to study the cost-effectiveness of universal UC CBC and the long-term outcomes of infants diagnosed with EO-NTCP.

## Introduction

Neonatal thrombocytopenia (NTCP) is a hematological condition characterized by platelet counts that are below the normal limit (<150 × 10^9^/L) [1]. It can be classified as mild (100-150 × 10^9^/L), moderate (50-99 × 10^9^/L), and severe (<50 × 10^9^/L) [2]. NTCP can be further classified based on the time of onset. For example, early-onset occurs within the first 72 hours of birth. Meanwhile, late-onset develops after 72 hours [3]. NTCP can be attributed to either immune or non-immune causes [4,5]. Immune causes include primary immune thrombocytopenia and neonatal autoimmune thrombocytopenia, and non-immune causes include chromosomal abnormalities and maternal factors, such as severe hypertension [4,5]. In severe cases, NTCP can lead to serious complications, such as perinatal intracranial hemorrhage, if left untreated [6].

Similar to other diseases such as early-onset anemia and neutropenia, NTCP can be detected using an umbilical cord complete blood count (UC CBC). In this procedure, blood samples are collected from the UC immediately after delivery. This procedure has several benefits. That is, it decreases the exposure to complications caused by invasive blood collection procedures among neonates, prevents unnecessary pain among newborns, and saves time and resources [7].

Previous studies have evaluated the possibility of replacing neonatal CBC with UC CBC [7-9]. However, these studies commonly focused on either preterm neonates or screening of full-term infants for sepsis [7-9]. Further, UC CBC was used on a per-risk basis, and no significant differences were found in terms of platelets, white blood cells (WBCs), hematocrit counts, and hemoglobin levels [7,8]. To the best of our knowledge, the use of UC CBC as a universal screening tool is not recommended by any accredited institution. However, some centers, including King Abdulaziz Medical City (KAMC), have used UC CBC based on local experience and unpublished data. If the use of UC CBC is approved, exposure to unnecessary blood collection procedures among newborns can be limited, thereby decreasing the risk of iatrogenic blood loss.

This study's aim was to evaluate the accuracy of UC CBC in the early detection of early-onset neonatal thrombocytopenia (EO-NTCP). Further, the prevalence of NTCP among patients admitted to KAMC, Jeddah, Saudi Arabia, was evaluated.

## Materials and methods

Study design, settings, and participants

This study used a cross-sectional design with a matched control. It was conducted for three consecutive years to evaluate the accuracy of UC CBC in detecting EO-NTCP and measure the prevalence of this disease. Additionally, the outcomes of EO-NTCP, including morbidity and mortality rates at KAMC in Jeddah, Saudi Arabia, were evaluated. KAMC is a tertiary care center in the city, with approximately 3,500 deliveries annually. The participants were selected from the postnatal wards, delivery suites, neonatal intensive care units (NICUs), and nurseries.

All live neonates (singleton and multiple, regardless of delivery type and gestational age) with routine non-clotted UC CBC samples at KAMC between May 2016 and May 2019 were included in the analysis. Meanwhile, neonates with congenital anomalies (such as skeletal dysplasia, major brain anomalies, and major heart diseases), whether diagnosed antenatally or postnatally, those with chromosomal abnormalities, and any fetus with immune or non-immune hydrops fetalis were excluded from the study.

The non-probability consecutive sampling technique was used in this study, and the participants were categorized into two groups. Based on the UC CBC results, the first group (cases) comprised newborns with EO-NTCP. The second group included neonates with normal platelet counts (controls) randomly selected in a 1:2 ratio. The selected controls were matched with cases based on sex, gestational age, and birth year.

Data collection process

Data were extracted from the electronic medical records system at KAMC (BestCare2.0, BESTCare System, Seoul, South Korea) and recorded in the data collection sheets. The following data were obtained: maternal variables (age, gravidity, parity, abortion, body mass index (kg/m²), antenatal care (ANC), intrauterine growth restriction (IUGR), and pregnancy-induced diseases such as gestational hypertension (GHTN), pre-eclampsia (PET), gestational diabetes (GDM), and immune diseases including immune thrombocytopenic purpura (ITP) and systemic lupus erythematosus (SLE)); delivery variables (mode of delivery, group B Streptococcus infection (GBS), oligohydramnios, umbilical cord potential of hydrogen (PH), and perinatal events, such as antepartum hemorrhage, fetal distress, ruptured placenta, and asphyxia/hypoxic-ischemic encephalopathy); and neonatal variables (sex, birth weight, gestational age, NICU admission, need for mechanical ventilation in the NICU, antibiotics taken in the NICU, neonatal sepsis, minor congenital anomalies, platelet or blood transfusion, length of hospital stay, final diagnosis of EO-NTCP, and mortality).

Data analysis

The prevalence of NTCP was calculated with respect to total life birth. The sensitivity, specificity, negative predictive value (NPV), and positive predictive value (PPV), and their 95% confidence interval (95% CI) of UC CBC were calculated as per definition using standard 2x2 table via MedCalc Statistical Software Version 22.014 (MedCalc Software Ltd., Ostend, Belgium) [10].

In the descriptive analysis, continuous variables were presented as mean and standard deviation or median and interquartile range (IQR) based on the normality assumption. Categorical variables were expressed as frequencies and percentages. The independent samples t-test or Mann-Whitney U test was used to evaluate continuous variables. Meanwhile, the chi-square or Fisher’s exact test was applied to assess categorical data. A p-value of <0.05 was considered statistically significant. All data were analyzed using IBM SPSS version 28 for Windows (IBM Corp., Armonk, NY).

Ethical consideration

This study was approved by the King Abdullah International Medical Research Center (protocol number: SP20/049/J). The need for an informed consent form was waived as data were collected via a chart review.

## Results

Prevalence of neonatal thrombocytopenia

**Figure 1:**
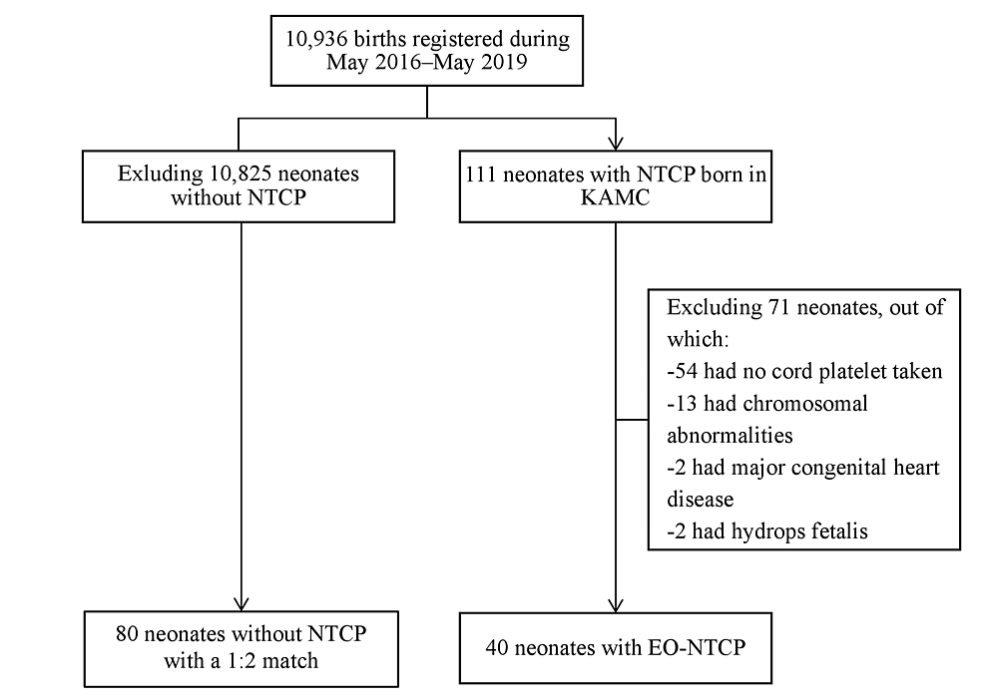
Diagram of patient selection. NTCP: neonatal thrombocytopenia, KAMC: King Abdullah Medical City, EO-NTCP: early-onset neonatal thrombocytopenia.

As shown in Figure 1, of 10,936 neonates born at KAMC between May 2016 and May 2019, 111 presented with NTCP, hence a prevalence of 10.15 per 1000 live births (1.02%). 

Characteristics of mothers

Among 111 neonates diagnosed with NTCP, 71 were excluded from the analysis (Figure 1). In total, 40 neonates underwent UC CBC, and 80 healthy neonates were considered as controls. As shown in Table 1 (A), 12 (30%) mothers without ANC gave birth to neonates with EO-NTCP, and 10 (12.5%) mothers without ANC gave birth to neonates without EO-NTCP (p = 0.02). IUGR was detected in 15 (37.5%) neonates with EO-NTCP and 5 (6.3%) neonates without EO-NTCP (p < 0.001). Five (12.5%) mothers who gave birth to neonates with EO-NTCP had oligohydramnios, and none of the mothers of neonates without EO-NTCP experienced oligohydramnios (p = 0.003). However, there was no statistically significant difference in terms of maternal age, gravidity, parity, abortion, body mass index, type of delivery, pregnancy-induced diseases, history of immune or renal diseases, GBS status, perinatal events smoking, and umbilical cord pH between the sample and control groups.

Demographic characteristics of the neonates

As shown in Table 1 (B), 34 (85%) neonates with EO-NTCP were admitted to the NICU, and 22 (55%) were exposed to antibiotics. On the other hand, 35 (43.8%) neonates without EO-NTCP were admitted to the NICU, and 25 (31.3%) of them were exposed to antibiotics (p < 0.001 and p = 0.012, respectively). Seven (17.5%) neonates with EO-NTCP were diagnosed with neonatal sepsis (either with positive blood or cerebrospinal fluid culture, or clinically with biochemical changes). Meanwhile, only three (3.8%) neonates without EO-NTCP had neonatal sepsis (p = 0.015). Fifteen (37.5%) neonates with EO-NTCP and 1 (1.3%) neonate without EO-NTCP received platelet transfusion (p < 0.001).

The median length of hospital stays (IQR) among neonates with EO-NTCP and those without were 13 (3-28) days and 4 (2-9) days (p = 0.006), respectively. The mortality rates of neonates with EO-NTCP and those without were 6 (15%) and 2 (2.5%) (p = 0.016), respectively. However, no statistically significant difference was found between the two groups regarding sex, birthweight, gestational age, minor congenital anomalies, need for mechanical ventilation in the NICU, and blood transfusion. The final diagnosis of neonates with EO-NTCP was as follows: 20 (50%) neonates were diagnosed with transient NTCP due to prematurity or pre-eclampsia, 18 (45%) with unspecified transient NTCP, 1 (2.5%) with alloimmune NTCP, and 1 (2.5%) with autoimmune NTCP.

**Table 1:** Maternal and neonatal variables. N (%). *Mean (SD). **Median (IQR). IQR: interquartile range, BMI: body mass index, kg/m^2^: kilogram per meter squared, ANC: antenatal care, GDM: gestational diabetes mellitus, GHTN: gestational hypertension, IUGR: intrauterine growth restriction, GBS: group B Streptococcus, g: gram, NICU: neonatal intensive care unit, LOS: length of hospital stay, SD: standard deviation.

Variables	Sample group (n = 40)	Control group (n = 80)	p-value
(A) Maternal variables			
Age*	29.6 (6.5)	29.2 (5.9)	0.8
Gravidity**	2 (2–4)	3 (2–4)	0.8
Parity**	1 (1–3)	1 (0–3)	0.8
Abortion**	0 (0–0)	0 (0–0.25)	0.9
BMI (kg/m^2^)*	28.8 (6.4)	29.1 (6.7)	0.8
Lack of ANC	12 (30%)	10 (12.5%)	0.02
Pregnancy-induced diseases			
None	26 (65%)	57 (71.3%)	0.2
GDM	5 (12.5%)	14 (17.5%)	
GHTN	9 (22.5%)	9 (11.3%)	
Renal diseases during pregnancy	0 (0%)	1 (1.3%)	1
History of immune diseases during pregnancy	3 (7.5%)	3 (3.8%)	0.4
Smoking (yes)	1 (2.5%)	0 (0%)	0.3
IUGR	15 (37.5%)	5 (6.3%)	<0.001
Cesarean section	20 (50%)	37 (46.3%)	0.7
GBS	6 (15%)	15 (18.8%)	0.6
Perinatal events	4 (10%)	12 (15%)	0.6
Oligohydramnios	5 (12.5%)	0 (0%)	0.003
Umbilical cord pH*	7.2 (0.10)	7.2 (0.13)	0.8
(B) Neonatal variables			
Sex (male)	29 (72.5%)	57 (71.3%)	0.9
Birth weight (g)*	2337.9 (1020.7)	2588.1 (761.6)	0.1
Gestational age (weeks)*	35.7 (4.8)	36.4 (3)	0.3
Admission to the NICU	34 (85%)	35 (43.8%)	<0.001
Need for mechanical ventilation in the NICU	15 (37.5%)	25 (31.3%)	0.5
Antibiotics taken in the NICU	22 (55%)	25 (31.3%)	0.012
Neonatal sepsis	7 (17.5%)	3 (3.8%)	0.015
Minor anomalies	7 (17.5%)	11 (13.8%)	0.6
Platelet transfusion	15 (37.5%)	1 (1.3%)	<0.001
Blood transfusion	3 (7.5%)	2 (2.5%)	0.3
LOS**	13 (3–28)	4 (2–9)	0.006
Mortality	6 (15%)	2 (2.5%)	0.016

Laboratory parameters

As shown in Table 2, after evaluating the burden of EO-NTCP during the first year of life, neonates with EO-NTCP required significantly more laboratory tests than those without EO-NTCP (p < 0.001). However, the inpatient, outpatient, and emergency room admission rates were similar between neonates with and without EO-NTCP. Table 3 presents the laboratory parameters of UC and follow-up infant CBC. Other than platelet count, which was low in both UC CBC and follow-up CBC in neonates with EO-NTCP, the laboratory parameters did not significantly differ between the sample and control groups. There was a difference in cord hemoglobin level between UC CBC and follow-up CBC. However, it was not clinically significant as it followed the normal distribution of expected hemoglobin levels without extreme values. 

**Table 2:** The burden of EO-NTCP. Median (IQR). IQR: interquartile range. EO-NTCP: early-onset neonatal thrombocytopenia.

Variables	Sample group (n = 40)	Control group (n = 80)	p-value
In-patient admissions	1 (1–1)	1 (0–1)	0.3
Emergency room admission	0 (0–1)	0 (0–1)	0.9
Outpatient admission	5 (0.25–9)	3 (1–7)	0.3
Laboratory examination	36 (16–69)	16 (7–25)	<0.001

**Table 3:** Laboratory parameters. Mean (SD). CBC: complete blood count, WBC: white blood cells, Hb: hemoglobin, SD: standard deviation.

Variables	Sample group (n = 40)	Control group (n = 80)	p-value
Cord CBC variables			
WBC count	9.5 (3.6)	9.5 (3.3)	0.9
Hb count	15.7 (2.4)	14.6 (2.2)	0.01
Hematocrit count	49.5 (7.9)	47.1 (9.9)	0.2
Platelet count	148.7 (81.8)	275.8 (73.9)	<0.001
Follow-up CBC variables (within three days)			
WBC count	11.1 (4.8)	11.8 (4.3)	0.4
Hb count	17.6 (3.7)	17.3 (2.3)	0.6
Hematocrit count	54 (11.3)	53.5 (11.4)	0.8
Platelet count	132.4 (76.6)	279.5 (81.4)	<0.001

Sensitivity and specificity of UC CBC

Table 4 measures the sensitivity, specificity, PPV, and NPV by comparing the platelet counts in UC CBC and follow-up CBC. The sensitivity and specificity of UC CBC in detecting EO-NTCP were 62.50% (95% CI: 45.80-77.27) and 97.50% (95% CI: 91.26-99.70), respectively. Using an estimated prevalence of 1.015%, the PPV and NPV of UC CBC were 20.40% (95% CI: 6.01-50.70) and 99.61% (95% CI: 99.41-99.74), respectively.

**Table 4:** Sensitivity, specificity, PPV, and NPV. *These values were based on disease prevalence. CBC: complete blood count, CI: confidence interval, PPV: positive predictive value, NPV: negative predictive value.

Cord CBC	Follow-up CBC (within 72 hrs)		Accuracy calculations			
	Positive	Negative	Sensitivity	Specificity	PPV*	NPV*
Positive	25	2	62.50% (95% CI: 45.80%–77.27%)	97.50% (95% CI: 91.26%–99.70%)	20.40% (95% CI: 6.01%–50.70%)	99.61% (95% CI 99.41%–99.74%)
Negative	15	78				

## Discussion

In this single-centered study, the prevalence of NTCP among newborns was approximately 1.02%. After reviewing local data, only one prospective observational study was performed in Riyadh between May and October 2013. Results showed that the incidence rate of NTCP within five months was 1.9% (84/4379), which is consistent with our study [2]. By contrast, several studies conducted in different countries have shown that the prevalence can vary in various populations [11-13]. Nevertheless, most international studies have reported that the prevalence of NTCP ranges from 1% to 5% [3,11,13-17]. However, the prevalence is significantly high among neonates admitted to the NICU [12,13,18]. In a retrospective study performed in Turkey between January 2007 and December 2011, the prevalence rates of NTCP were 3.8% among all hospitalized newborns and 12% among preterm infants [11]. In addition, a prospective multicenter study was performed in Paris, and the results showed that the 16-month prevalence rate was approximately 0.9%. However, in the study, a large group of patients were excluded due to a significantly low birth weight, which could have made the sampling difficult, ultimately leading to inaccurate results [12]. They theorized that the unscreened group had a higher number of newborns who were small for their gestational age [12]. In 1998, a large-scale study with 8388 newborns performed in Switzerland showed that 0.5% of patients presented with NTCP [19]. Notably, the prevalence of NTCP might have increased in recent years, which warrants further investigation.

Some coexisting maternal characteristics were found to be risk factors for giving birth to neonates with EO-NTCP. Among them is the absence of ANC during pregnancy, which affects early detection of the disease. Mothers without ANC (30%) were more likely to give birth to babies with EO-NTCP. Moreover, mothers with IUGR during pregnancy (37.5%) had a higher risk of giving birth to neonates with EO-NTCP. Some of these factors were similar to those found in the literature. For example, two studies conducted in 2013 and 2016 reported that IUGR and septicemia were the most common causes of early- and late-onset NTCP, respectively [11,20]. Another risk factor identified in our study was oligohydramnios. That is, 12.5% of mothers with oligohydramnios gave birth to neonates with EO-NTCP. A 2016 study conducted in India reported that oligohydramnios was a maternal risk factor for NTCP. The study stated that 8 of 200 mothers with oligohydramnios gave birth to babies with NTCP [15]. 

Additionally, this study has found that neonates with EO-NTCP have less favorable outcomes as they are more likely to be admitted to the NICU (85%) and take antibiotics (55%). According to the literature, 18%-35% of neonates admitted to the NICU develop NTCP, and the incidence rate might vary based on the selected sample's gestational age and birth weight [11]. In our study, EO-NTCP was associated with other outcomes, such as sepsis (17.5%) and a high mortality rate (15%). A 2018 local study in Riyadh reported that the mortality rate of patients with NTCP was 3.5%. However, several international studies have shown that the mortality rate can range from 1% to 10% [2,21,22]. This could be explained by the fact that our institution is a tertiary referral center for high-risk pregnancies associated with IUGR anomalies and oligohydramnios. Further, neonates with EO-NTCP were more likely to receive platelet transfusions (37.5%). A previous study found that 45 of 134 (33%) neonates with NTCP received platelet transfusions [11]. Other studies have revealed that 25% of newborns with platelet counts of <150 × 10^9^/L received at least one transfusion, and this percentage increases with extremely low birth weight (<1000 g) and low platelet counts [16].

According to our results, UC CBC is highly specific (97.5%) but not sensitive (62.5%) in detecting EO-NTCP. Therefore, this test can help physicians discharge patients with normal cord platelet counts with high confidence without the need to repeat CBC. However, a positive cord result cannot be used entirely for disease diagnosis, and follow-up is required for confirmation. Considering the low prevalence of NTCP, a high NPV indicates that UC CBC can be safely used to reassure parents of neonates with negative results, thereby supporting our current practice of not repeating CBC among healthy neonates. A poor PPV, however, indicates the need for a comprehensive clinical history and physical examination with a confirmatory follow-up infant CBC to validate the diagnosis. 

The accuracy of UC CBC regarding the diagnosis of EO-NTCP was not previously evaluated. However, some reports compared laboratory parameters of UC and follow-up CBC. For instance, a study comprising 100 late preterm infants was performed in New York, USA, 2017. Paired cord and follow-up blood samples were compared regarding hemoglobin level and WBC, absolute neutrophil, and platelet counts. The results showed no significant differences between the laboratory parameters measured in both samples. Therefore, UC CBC can be an alternative to admission CBC in late preterm infants. Nevertheless, the accuracy of UC CBC in detecting NTCP was not assessed [7]. Similarly, in 2012, a previous study using 174 paired cord and admission blood samples collected from infants with a gestation age of <35 weeks was conducted. Results showed a significant correlation between the hemoglobin level and platelet count in UC CBC and admission CBC. Thus, they reported that UC CBC can be an alternative method [8]. Another cohort study assessed the correlation between paired UC and infant blood samples regarding immature-to-total granulocyte ratio and WBC, hematocrit, and platelet counts in sepsis screening for 113 asymptomatic term infants [9]. This study reported a significant correlation between the two samples concerning the aforementioned parameters; thus, they concluded that cord blood was a safe alternative for infant blood in routine sepsis evaluations in asymptomatic neonates [9].

To the best of our knowledge, this study is the first to assess the sensitivity and specificity of UC CBC, specifically in diagnosing EO-NTCP in both term and preterm infants. Moreover, this study contributed to reducing the gap in local data about the prevalence of NTCP. Furthermore, a sample group was matched with a control group regarding gestational age, sex, and birth year. This reduced the impact of confounding factors, resulting in a relatively balanced comparison.

The current study had some limitations. That is, there were missing data, which could not be controlled due to the study's retrospective nature. In addition, the relatively small sample size could affect the results' accuracy. Finally, as follow-up was only conducted within 72 hours, our results applied to EO-NTCP alone. Further risk factor analysis must be undertaken to examine the effects of antenatal determinants using the antenatal risk score and multi-domain, validated socioeconomic status [23,24]. Long-term follow-up is recommended for infants with NTCP to assess healthcare utilization and neurodevelopmental outcomes [25,26]. To overcome the above-mentioned limitations, a multicenter study must be performed in the future. Also, since those with chromosomal anomalies were excluded, we suggest to extend the research to involve a chromosomal analysis in the future.

## Conclusions

The prevalence of EO-NTCP in KAMC is comparable to international and national figures, and it is associated with preceding maternal comorbidities, serious neonatal morbidity, and even mortality. Therefore, proper ANC is vital in preventing maternal and neonatal morbidities, including the risks of NTCP and its related complications. With high NPV, using UC CBC as a universal screening method could assist in safely discharging newborns. However, because of its low sensitivity, a comprehensive clinical examination with confirmatory laboratory tests are still the cornerstone in diagnosing EO-NTCP. Future trials should aim to study the cost-effectiveness of universal UC CBC and the long-term outcomes of infants diagnosed with EO-NTCP. 
